# Free-breathing 3D whole-heart coronary mra using respiratory motion-resolved sparse reconstruction

**DOI:** 10.1186/1532-429X-18-S1-O105

**Published:** 2016-01-27

**Authors:** Davide Piccini, Li Feng, Gabriele Bonanno, Simone Coppo, Jérôme Yerly, Ruth P Lim, Juerg Schwitter, Daniel K Sodickson, Ricardo Otazo, Matthias Stuber

**Affiliations:** 1 Advanced Clinical Imaging Technology, Siemens Healthcare, Lausanne, Switzerland; 2 Department of Radiology, University Hospital (CHUV) and University of Lausanne (UNIL), Lausanne, Switzerland; 3 Department of Radiology, Center for Advanced Imaging Innovation and Researc (CAI2R) and Bernard and Irene Schwartz Center for Biomedical Imaging, New York, NY USA; 4 grid.433220.4Center for Biomedical Imaging (CIBM), Lausanne, Switzerland; 5 grid.1008.9000000012179088XDepartment of Radiology, Austin Health and The University of Melbourne, Melbourne, VIC Australia; 6 grid.8515.90000000104234662Division of Cardiology and Cardiac MR Center, University Hospital of Lausanne (CHUV), Lausanne, Switzerland

## Background

Navigator gating is commonly used to minimize respiratory motion in free-breathing whole-heart coronary MRA [1]. However, lengthy and unpredictable acquisition times remain a drawback. Respiratory self-navigation (SN) [2-3], conversely, enables 100% scan efficiency, but performs motion correction over a broad range of respiratory displacements, which can result in image artifacts. Here, we propose an alternative respiratory motion-resolved approach based on 3D radial phyllotaxis sampling, respiratory motion sorting and sparse reconstruction.

## Methods

**Figure 1:**
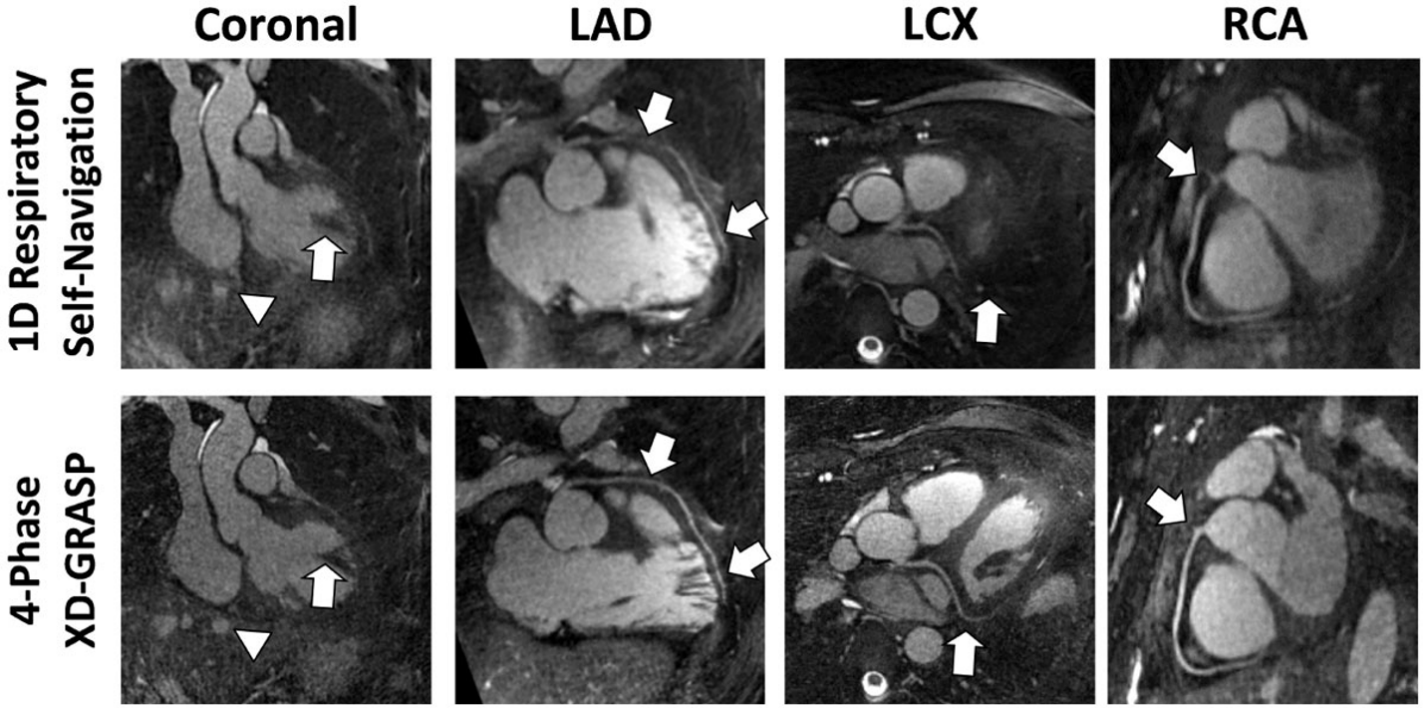
**Example of results showing the improvements (arrows) between 1D self-navigation and XD-GRASP reconstruction**.

Examinations in N = 11 healthy volunteers (9 male, age: 29 ± 4 y) were performed on a 1.5T clinical MRI scanner (MAGNETOM Aera, Siemens Healthcare) with a prototype 3D radial phyllotaxis bSSFP sequence [4]: TR/TE 3.1/1.56 ms, FOV (220 mm)^3^, matrix 192^3^, voxel (1.15 mm)^3^, RF angle 115°, and receiver BW 898 Hz/Px. Using a respiratory signal directly extracted from the modulations of the k-space center amplitude within the radial imaging data [5], signal-readouts were grouped according to the respiratory state at which they were acquired (Fig. 1). The resulting series of undersampled respiratory states were reconstructed using an eXtra-Dimensional Golden-angle RAdial Sparse Parallel imaging (XD-GRASP) [6] algorithm, which exploits sparsity along the newly created respiratory dimension. Datasets for 4 respiratory states were reconstructed. Image quality of the end-expiratory phase was compared to 1D respiratory self-navigation in terms of vessel sharpness (VS) [7], visible length (VL) and diagnostic quality on a scale from 0 (non-visible) to 2 (diagnostic).

## Results

Respiratory-resolved XD-GRASP reconstruction effectively suppresses respiratory motion artifacts (Fig. 1). Average VS and VL were always superior for the respiratory-resolved datasets, reaching statistical significance (p < 0.05) for the left main (LM), for the proximal and mid left anterior descending artery (LAD) (e.g. VS of mid LAD 40.8 ± 9.1% vs 34.9 ± 10.2%) and for the mid right coronary artery (RCA). Visualized length of LM+LAD was significantly increased as well. A total of 41/88 coronary segments were graded as diagnostic for 1D SN, while this ratio increased to 61/88 for the XD-GRASP reconstruction (Tab.1). The XD-GRASP reconstruction reached 100% diagnostic quality for LM, proximal-LAD, and proximal-RCA.

## Conclusions

Instead of discarding data or enforcing motion models for motion correction, XD-GRASP makes constructive use of all respiratory phases to improve image quality, and achieves superior quality compared to 1D respiratory SN without the need for breath-holding, navigators, or complex 3D respiratory motion correction schemes. The phyllotaxis trajectory and XD-GRASP reconstruction provide a synergistic combination that may lead routine coronary MRA closer to clinical practice.

**Table 1:** Diagnostic quality grading of all coronary segments

Coronary Segment	1D Respiratory Self-Navigation	4-Phase X-D GRASP (End-exp)
Left Main	1.8 ± 0.4	2.0 ± 0.0*
LAD Prox.	1.6 ± 0.5	2.0 ± 0.0*
LAD Mid	1.3 ± 0.6	1.4 ± 0.5
LAD Dist.	0.9 ± 0.5	1.3 ± 0.5
LCX Prox.	1.4 ± 0.7	1.4 ± 0.7
RCA Prox.	1.8 ± 0.4	2.0 ± 0.0
RCA Mid	1.3 ± 0.5	1.7 ± 0.5
RCA Dist.	1.4 ± 0.7	1.7 ± 0.5
Total Diagnostic Segments	41/88 (47%)	61/88 (70%)

All values are expressed as mean ± one standard deviation

* Indicates statistical significance compared to 1D Respiratory Self-Navigation.

Diagnostic Grading: 0 = non-visible, 1 = visible but non diagnostic and 2 = visible and diagnostic

